# Supplementary figures and images for: Genetic links between endometriosis and cancers in women
Source: PeerJ. 2019 Dec 20;7:e8135. doi: 10.7717/peerj.8135 (PMC6927350; doi:10.7717/peerj.8135)

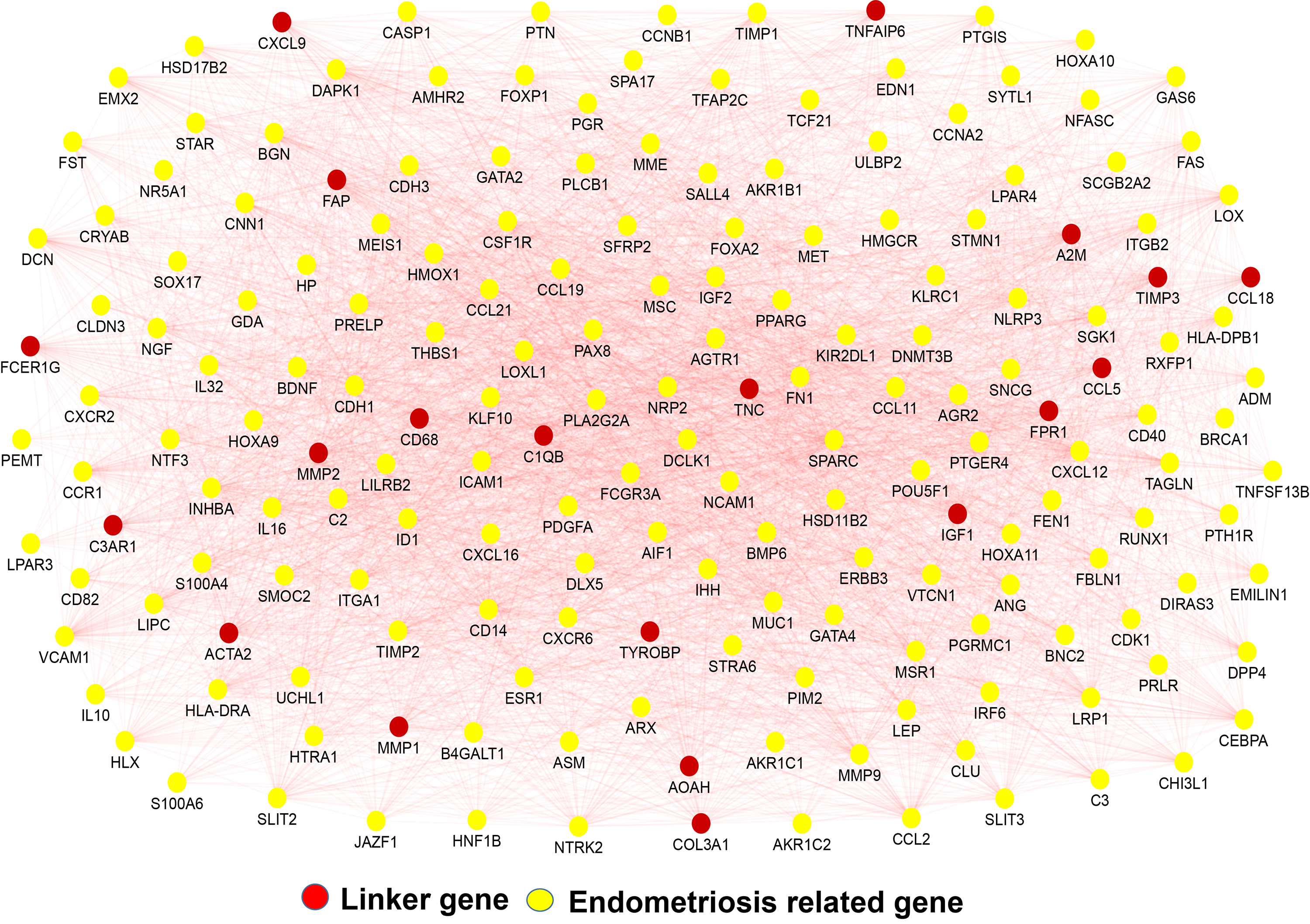

Supplement: Figure S1 — The network represents the molecular function-based relationship between these genes and the novel linker genes in cancer development. Yellow circles represent endometriosis-related genes, and red circles indicate linker genes. [file peerj-07-8135-s001.png]

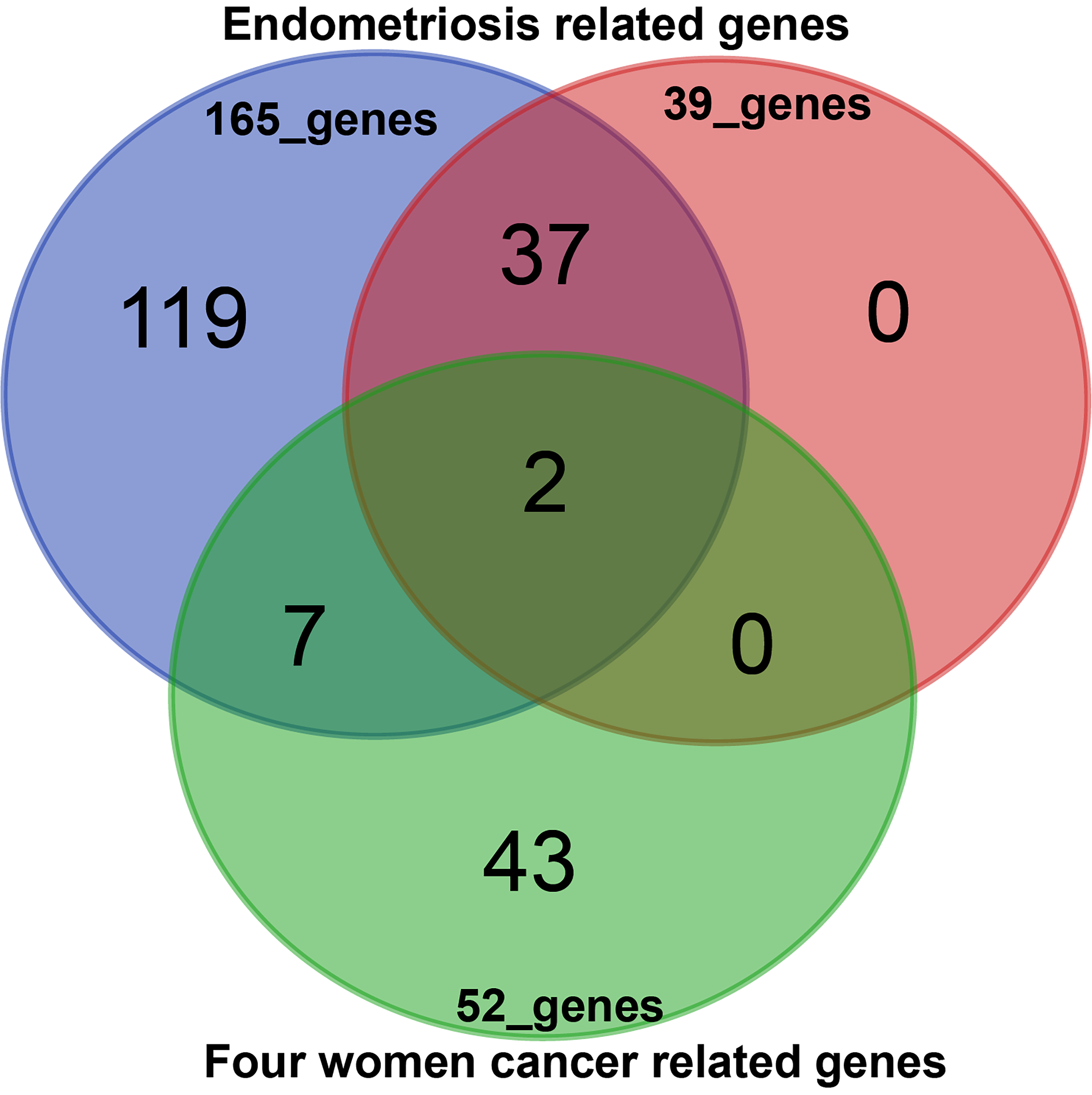

Supplement: Figure S2 — 165 (39 + 65 + 61) genes shared at least one GEO dataset, 39 genes shared between two GEO dataset and 52 genes common in four women specific cancer. [file peerj-07-8135-s002.png]
